# Special Issue: Rabies Symptoms, Diagnosis, Prophylaxis, and Treatment

**DOI:** 10.3390/tropicalmed2040059

**Published:** 2017-11-14

**Authors:** Charles E. Rupprecht, Bernhard Dietzschold

**Affiliations:** 1 LYSSA LLC, Cumming, GA 30040, USA; 2 Department of Microbiology and Immunology, Thomas Jefferson University, Philadelphia, PA 19107, USA; Bernhard.Dietzschold@jefferson.edu

Rabies is an acute, progressive, incurable viral encephalitis found throughout the world. Despite being one of the oldest recognized pathogens, its impact remains substantial in public health, veterinary medicine, and conservation biology. Thus, it is essential to apply existing tools and to seek new methods to improve upon prevention, control, selective variant elimination, and treatment efforts. Advances in diagnosis, vaccinology, pathobiology, and related research techniques continue to afford enhanced insights on rabies. Although rabies is not a candidate for eradication, the results of these innovative communications provide further knowledge to define a more optimal approach to understanding and managing this complex infectious disease of nature on a global basis in a One Health context.

More than 24 papers have been published upon peer review acceptance in this special issue (20 original papers, 1 perspectives piece, and 4 review papers are included). They each contribute to a much better understanding of this disease and to advances concerning the improvements for rabies management. These topics can be summarized as follows:

The clarion call for action was sounded in a perspective by David Durrheim that provides an ideal framework for the ongoing tragedy exemplified by childhood deaths from rabies and application of the necessary steps to end this situation now [1]. Such a thoughtful piece is made all the more enigmatic considering the breakthroughs that have occurred over the millennia, as reviewed by Tarantola [2] and which appear to remain somewhat complicated in practice or ignored in diverse and disparate regions, such as across Asia and in the Caribbean, as reviewed by Buchy et al. [3] at a continental focus, and Seetahal et al. [4] locally for Trinidad (where the unique appreciation of rabies in vampire bats was one of the first examples to be documented and investigated in the New World), respectively.

One underlying theme is paramount: the key to reducing human rabies deaths is the mass vaccination of dogs, which serve as the major global reservoir responsible for the substantial public health burden today. A much better appreciation of the human animal bond, with a focus upon pet prophylaxis as the primary strategy to overcome many public health impacts of rabies, is supported by the work of Palamar et al. [5]. All developed countries have eliminated canine-transmitted rabies. Increasingly, developing countries have also achieved this success, especially in the New World. However, even in North America, reintroduction from abroad or via wildlife is a concern, with a feasible solution for free-ranging dogs in distinct communities such as the Navajo nation, as described by Bender et al. [6]. Additionally, uncontrolled foci at affected borders remain a threat as long as rabies remains in a region, as evidenced in the account from Peru by Castillo-Neyra et al. [7]. Foci remain within Central and South America, as well as the Caribbean. In Haiti, the country most affected by rabies in the New World, Medley et al. [8] present a concept of applied risk assessments combined with a laboratory-based diagnostics focus upon protocols to ensure that exposed individuals receive the needed prophylaxis in a resource-limited environment. In the same vein in the Old World, Lechenne et al. [9] for Chad and Coetzer et al. [10] for Lesotho, discuss the utility of surveillance and control by mass vaccination of dogs as a critical component for relief of the human rabies burden. Such focal projects there and elsewhere demonstrate the need for a pan-African approach, as championed by Pieracci et al. [11].

As described in the above communications, human rabies may be prevented and dog rabies can be eliminated. However, cross-species transmission complicates what appears to be a somewhat simple system. All warm-blooded vertebrates are susceptible to infection. Beyond dogs, meso-carnivores also act to perpetuate the disease in the Americas, Eurasia, and Africa. Perhaps uniquely among the zoonoses, vaccination against rabies can be applied to such free-ranging populations. In fact, western Europe is largely free of rabies by oral vaccination of red foxes and raccoon dogs. Similar successful programs are operative in North America against gray foxes and coyotes. Subjectively, the meso-carnivore species of greatest concern in Canada and the USA is the raccoon (*Procyon lotor*), as discussed by the following series of related papers. Kirby et al. [12] describe a system for enhanced surveillance of raccoon rabies in the eastern USA. Slate et al. [13] present the data on the use of a tiered system of suspect animals and index of activity centered upon road-killed raccoons. In concert, the use of a decentralized enhanced laboratory-surveillance system using a direct rapid immuno-histochemical test contributes not only to a highly sensitive and specific method concentrated on suspect wildlife in the USA, but also in Canada, as described for a new focus of raccoon rabies in southern Ontario, by Middel et al. [14]. Using the information from public health and wildlife rabies detection, Algeo et al. [15] formulated a model to track raccoon rabies spread over landscape corridors, as an approach to understanding its epizootiology and management from the aerial distribution of vaccine-laden baits. This method is efficient across broad areas but cannot be used easily in urban and suburban ecosystems. Hence, bait stations may prove useful in reaching these distinct raccoon populations, as described in Massachusetts and Florida by Bjorkland et al. [16] and Haley et al. [17].

Besides rabies virus, at least 15 other lyssaviruses cause this disease and more are expected for additional pathogen discovery. Cross-reactivity for all veterinary and human rabies vaccines may be limited against some of these lyssavirus species. To this effect, Kgaladi et al. describe an experimental approach to develop a panlyssavirus vaccine [18]. In addition to prevention or control concerns, relatively little is understood about the pathobiology of these diverse lyssaviruses in their various hosts, such as bats. Suu-Ire et al. describe the results from experimental infection of bats to one major lyssavirus, Lagos bat virus [19]. Similarly, when routine surveillance of wildlife is lacking, enhanced detection may be needed to augment a description of regional lyssavirus reservoirs. Virus neutralizing antibodies are one of the most critical immune effector products in vaccine-mediated immunity in all studied species, regardless of administration route, as well as in abortive infection, but the dilemma in drawing firm conclusions about absolute sero-protection dynamics among wildlife from investigations to date is summarized by Moore et al. [20]. Regarding serology, Tyem et al. utilized sero-surveillance in bat populations to fill in such gaps [21]. However, because many bats are small-bodied, limits to blood volume collection may be a liability for such surveys. Smith and Gilbert [22] describe a micro-neutralization test that can help overcome such issues for focal serological work in laboratory and field applications. 

The blood–brain barrier is a formidable concern when trying to deliver certain biologics to the CNS, especially in the treatment of encephalitis. To this end, data on further technological improvement for the use of a highly attenuated rabies virus recombinant vaccine in disease prevention and potential treatment is offered by Lebrun et al. [23]. Needs for improved passive immunity via alternative methods to polyclonal immune globulins, such as monoclonal antibodies (MAb), were first described at the end of the 1970s. Since then, numerous studies have shown the utility of these products as a potential replacement for rabies immune globulin. To this effect, another example of some of the epidemiological complexities posed by bat rabies virus variants, for a broadly reactive MAb candidate is discussed by Franka et al. [24].

Finally, Warrell et al. [25] can be understood in a similar context as the issue discussion began—rabies can be prevented by rapid and appropriate postexposure prophylaxis, but retains the title as the entity with the highest case fatality. As such, once clinical signs manifest, frustration and futility ensue for all involved. Rather than be treated as a pariah, at a very minimum, modern medicine offers palliation to the victim afflicted with this heinous affliction, as attempts for treatment continue. Towards this latter point, a challenge is presented to veterinarians at large to use their considerable time, talent and treasure to vaccinate all companion animals at risk and begin to develop safe and effective measures to treat clinical rabies as it presents in the domestic animals under their charge, given their oath and the biomedical tools at hand now, first by palliation at the very least and predictably by intervention. All veterinarians and their staff should be vaccinated, reliving a major concern from the unvaccinated physicians and nurses that care for rabies patients now. In this manner, a better collective approach will evolve for all species at hand, including *Homo sapiens*. Please recall that Pasteur, a biochemist, embarked on this controversial path from animal models to Joseph Meister and, at one time, canine vaccination was viewed as an unrealistic fantasy—it is far time the veterinary profession accepted the same responsibility towards the ‘incurable wound’ as true One Health demands.

In retrospect, the comparative number and the diversity of papers, the depth of the topics and the geographical reach of the authors from the Americas, Africa, Eurasia, and Australia in this special issue on rabies confirm the continued collective major interest in this area. This eclectic open access collection contributes to a much better understanding on the detection, prevention, control, selective elimination, and eventual therapy of this ancient zoonosis. We hope that you may enjoy digesting their content as much as we were pleased to share them with an international audience and look forward to future opportunities to broaden such success to the field at large. Optimistically, if even a single individual is inspired by a new thought in one of these manuscripts, then our objective is accomplished.
